# An integrated multimodal approach to drug repurposing in endometriosis, using ROR1 as a target

**DOI:** 10.3389/fphar.2025.1716062

**Published:** 2025-12-03

**Authors:** Kate Gunther, Dongli Liu, Gill Stannard, Melissa Holmes, Christine Loo, Belinda Guo, Nikola Bowden, Jason Abbott, Caroline E. Ford

**Affiliations:** 1 Gynaecological Cancer Research Group, Lowy Cancer Research Centre, School of Clinical Medicine, Faculty of Medicine and Health, UNSW Sydney, Sydney, NSW, Australia; 2 Gynaecological Research and Clinical Evaluation (GRACE) Unit, UNSW Sydney, Sydney, NSW, Australia; 3 Discipline of Women’s Health, School of Clinical Medicine, UNSW Sydney, Sydney, NSW, Australia; 4 National Endometriosis Clinical and Scientific Trials (NECST) Network, Sydney, NSW, Australia; 5 Consumer Working Group Leader, School of Medicine and Public Health, University of Newcastle, Sydney, NSW, Australia; 6 Histopath Diagnostic Specialists, Newcastle, NSW, Australia; 7 South Eastern Area Laboratory Services (SEALS), Sydney, NSW, Australia; 8 School of Medicine and Public Health, University of Newcastle, Newcastle, NSW, Australia; 9 Prince of Wales Private Hospital, Sydney, NSW, Australia

**Keywords:** endometriosis, drug repurposing, Ror1, organoid, reimegepant

## Abstract

**Background:**

Endometriosis is a chronic, heterogeneous disease with limited non-hormonal treatment options. Drug repurposing provides an accelerated route to identify safe, tolerable, and potentially effective therapies for endometriosis. Receptor tyrosine kinase-like orphan receptor 1 (ROR1) was investigated as a potential target based on its restricted expression in adult tissues and emerging role in the pathogenesis of multiple diseases.

**Methods:**

ROR1 expression was assessed in transcriptomic datasets including 408 endometriosis samples and 53 controls and validated at the protein level in an independent cohort of tissue microarrays comprising 179 tissues. Candidate compounds predicted to bind ROR1 were prioritized using the BLAZE platform, filtered for pharmacological safety and patient acceptability, and screened in the 12Z endometriotic epithelial cell line. The compound that showed the greatest reduction in proliferation and viability, rimegepant, was further tested in three patient-derived organoid models representing deep infiltrating endometriosis to evaluate viability, growth, and morphological responses.

**Results:**

ROR1 was transcriptionally upregulated in endometriosis and overexpressed at the protein level across lesions. Of three shortlisted compounds, cabergoline and pirenzepine did not alter proliferation, while rimegepant significantly reduced viability in 12Z cells. In patient-derived organoids, responses were patient-specific: two models showed concentration-dependent antiproliferative and cytotoxic effects, while one model was less responsive at the concentrations tested. Morphological features consistent with cell death were observed in sensitive lines.

**Conclusion:**

This study provides the first evidence in human-derived endometriosis models supporting rimegepant, a clinically approved calcitonin gene-related peptide antagonist with a favorable safety profile, as a potential therapy. The integrated pipeline combining molecular validation, computational prioritization, and patient-derived functional testing illustrates a translational approach to accelerate drug discovery in endometriosis.

## 1 Introduction

Endometriosis is a chronic and systemic condition involving immune, inflammatory, and fibrotic processes, defined by the growth of endometrial-like tissue outside the uterus. Affecting over 190 million people worldwide, it is linked to chronic pelvic pain, infertility, and other symptoms that profoundly disrupt daily life and wellbeing (World Health Organization, 2023). Despite its prevalence and impact, treatment options for endometriosis remain limited, with most therapies relying heavily on hormonal suppression, surgery or analgesia (Alonso et al., 2024). These approaches often offer only partial symptom relief, carry significant side effects, and are unsuitable for many patients (Pinheiro et al., 2023; Mitchell et al., 2023). There is a pressing need for non-hormonal, targeted therapies that address the underlying biology of the disease.

Drug repurposing is an attractive strategy in the search for new endometriosis treatments. This approach involves identifying new therapeutic uses for existing or previously studied agents, leveraging known safety and pharmacokinetic profiles to shorten development timelines and reduce costs (Pushpakom et al., 2019). Given the chronic nature of endometriosis, the limitations of hormonal therapies, and the disproportionate underfunding of endometriosis research compared to other diseases, drug repurposing represents a pragmatic and timely approach to accelerate access to novel, non-hormonal therapies with established safety profiles and real-world tolerability (Nikanjam et al., 2024).

Drug repurposing has a proven track record in oncology, where many targeted agents originally developed for one indication have been successfully repositioned for cancer treatment. One notable example is thalidomide, first introduced in the 1950s as an oral sedative with widespread use in pregnancy to reduce nausea. By 1961, it was withdrawn from the market due to confirmed links to teratogenicity (Vargesson and Stephens, 2021). In the mid-1960s, thalidomide was tested as a sedative for a severe form of treatment-resistant leprosy, erythema nodosum leprosum. The severe symptoms of erythema nodosum leprosum responded to thalidomide and it was quickly established as an effective treatment for the disease (Pearson and Vedagiri, 1969).

Almost 20 years later, thalidomide showed effectiveness for graft-vs-host disease (Lim et al., 1988) and extensive laboratory studies were undertaken to determine the mechanism of action for thalidomide. In 1994, 40 years after its first use as a sedative, an anti-angiogenic mechanism of action of thalidomide was reported (D’amato et al., 1994). Within the next 9 years, it was repurposed for relapsed and refractory multiple myeloma, a disease that previously carried complete remission rates below 5% and median survival under 3 years (Sporn and Mcintyre, 1986). As a repurposed therapy, thalidomide is now a cornerstone of multiple myeloma treatment, and when combined with other agents has an overall survival rate of up to 86% (Costa et al., 2022). This highlights how existing drugs can be rapidly redeployed to deliver transformative outcomes in diseases with few options.

Characterized by a paucity of expression in adult tissues yet overexpressed in multiple malignancies (John and Ford, 2022), receptor tyrosine kinase-like orphan receptor 1 (ROR1) has emerged as a target of interest in precision medicine, including gynecological cancers (Liu et al., 2025a; Liu et al., 2025b). ROR1 acts as a receptor tyrosine kinase-like protein that transduces non-canonical Wnt signaling upon binding its ligand, Wnt5a, modulating cellular processes such as proliferation, migration, and survival that support tumor growth and maintenance (Kipps, 2022). In endometriosis, evidence suggests ROR1 is upregulated in stromal cells of ovarian endometriomas through epigenetic mechanisms (Yotova et al., 2017), and Wnt5a contributes to stromal signaling and the immune microenvironment (Liu S. et al., 2025; Yang et al., 2023). Collectively, these observations highlight ROR1 as a biologically plausible target for therapeutic intervention in endometriosis, although its potential has yet to be experimentally validated.

This study aimed to validate ROR1 as a therapeutic target in endometriosis and to employ computational chemistry approaches to identify existing compounds with potential affinity for this receptor. To achieve these aims, a drug repurposing framework was applied, combining target validation with *in silico* screening to accelerate the identification of candidate non-hormonal treatments. The efficacy of the selected compounds was then tested using a 2D endometriosis cell line and 3D patient-derived organoid models, providing experimental validation of repurposed drug candidates for the treatment of endometriosis.

## 2 Methods

### 2.1 Gene expression analysis

Gene expression and associated phenotype data from endometriosis tissues were obtained from GSE141549. The dataset comprised expression data from 408 samples across 115 patients and 53 controls, including control endometrium (n = 37), eutopic endometrium (n = 102), control peritoneum (n = 24), endometriosis peritoneum (n = 37), superficial peritoneal endometriosis (n = 76), deep infiltrating endometriosis (n = 88), and endometrioma (n = 28). Samples were classified by hormonal treatment status within 3 months prior to surgery, and menstrual cycle phase was determined by histological dating performed by an experienced pathologist. This dataset was generated using microarray hybridization on Illumina HumanHT-12 V3.0 or V4.0 expression beadchips. Raw data had been processed by the original authors, including background correction, detection p-value filtering (p < 0.05), quantile normalization, and log_2_ transformation, as previously described (Gabriel et al., 2020). Expression data for the gene of interest, *ROR1*, were extracted. For 15 samples, multiple replicates were obtained from the same tissue specimen (i.e., same lesion or endometrial site). These included 2 patient peritoneum samples, 3 superficial endometriosis lesions, 3 deep infiltrating endometriosis lesions, 2 eutopic endometrium samples, and 5 control endometrium samples. Expression values for these replicates were averaged to generate a single representative value for each tissue sample.

### 2.2 Computational drug target identification

Drug target analysis was performed as previously described (Matthews et al., 2024). Briefly, compounds predicted to bind between the ROR1-Kringle domain and the antibody heavy chain variable domain of ROR1 were identified using the BLAZE™ ligand-based screening platform (Cresset Discovery Services UK). This platform employs molecular field-based similarity scoring to compare electrostatic and conformational properties of ligands against known compounds. Docking simulations generated affinity scores, with only compounds exceeding a predefined affinity threshold retained. The list was filtered to include compounds approved for human use by the United States Food and Drug Administration (FDA), European Medicines Agency (EMA), or Australian Therapeutic Goods Administration (TGA). Comprehensive literature searches were conducted using PubMed, Embase, DrugBank, and clinical trial registries up to October 2023, with search terms combining agent names and keywords such as “dosing,” “toxicity,” “pharmacokinetics,” “delivery,” “teratogenicity,” “cytotoxicity,” and “long-term safety”. Data were extracted on dosing ranges, toxicity profiles, delivery modes, lipophilicity, teratogenicity, and cytotoxicity. Compounds failing to pass phase I clinical trials, lacking commercial availability, or with significant safety concerns or pharmacological limitations were excluded. Priority was given to agents demonstrating minimal toxicity, oral bioavailability, documented safe long-term use, and low side-effect incidence. The top six candidates underwent independent review by a clinical expert (JA), focusing on safety in relevant comorbidities, and a consumer advocate (GS), focusing on treatment acceptability.

### 2.3 Two-dimensional cell culture

The 12Z endometriotic epithelial cell line (Sigma Aldrich, SCC443) was cultured according to the manufacturer’s instructions in DMEM/F12 medium supplemented with 10% fetal bovine serum (Scientifix, #FBSFR-SOOJF, Australia), 1% GlutaMAX (Gibco, #35050061, United States), and 1% penicillin-streptomycin (Gibco, #15140122, United States). Cells were maintained at 37 °C in a humidified atmosphere containing 5% CO_2_, and were tested routinely for *mycoplasma* contamination.

For spheroid formation, cells were seeded at a density of 4 × 10^4^ cells/mL in 100 µL volumes into the central 60 wells of ultra-low attachment 96-well plates (Corning, #7007, United States). Plates were incubated at 37 °C with 5% CO_2_ for 4 days to allow spheroid formation. Resulting spheroids were collected by centrifugation at 1,500 rpm for 5 min and resuspended in warmed HistoGel (Epredia, #22–110–678, United States). HistoGel domes were allowed to solidify at room temperature for 15 min, then fixed in 4% formalin for 1 hour before transfer to 70% ethanol for storage. Fixed HistoGel domes were embedded in paraffin and sectioned at 4 µm for subsequent staining procedures.

Western blot analysis was performed on a protein lysate from 12Z cells of the same passage as one of the triplicates used for the cell viability assay to confirm ROR1 expression (data presented in Supplementary Figure 2b).

### 2.4 Cell viability assays

12Z cells were seeded at 4 × 10^4^ cells/mL in 96-well plates (Corning, #3516, United States) and incubated for 24 h prior to treatment. Pre-treatment baseline images were captured using an IncuCyte live-cell imager (Sartorius). Test compounds were prepared as concentrated stocks in DMSO and diluted in complete DMEM/F12 or human plasma-like medium (HPLM, Gibco, #A4899101, United States), each supplemented with 10% fetal bovine serum (Scientifix, #FBSFR-SOOJF, Australia) and 1% penicillin-streptomycin (Gibco, #15140122, United States). Drug treatments were applied as serial dilutions ranging from 100 µM to 0.78 µM, ensuring a final DMSO concentration of ≤0.1% in all wells. Vehicle control wells contained 0.1% DMSO to match solvent concentration, while 25% DMSO was used as a positive control to induce cell death. Treatments were applied in triplicate wells. Plates were imaged every 4 h for 72 h.

At 72 h, 50 µL of MTT solution (Sigma-Aldrich, #M2128) at 0.5 mg/mL in PBS was added to each well. Plates were incubated for 3 h to allow formazan crystal formation. Media and MTT solution were carefully removed, and 100 µL of MTT solubilization solution (0.04 M HCl in absolute isopropanol) was added. Plates were shaken at 900 rpm for 5 min to dissolve crystals, and absorbance was read immediately on a SpectraMax 190 plate reader (Molecular Devices) at 570 nm with 630 nm background correction.

### 2.5 Establishment and maintenance of patient-derived organoids

Biospecimens and clinical data were collected under approval from the South Eastern Sydney Local Health District Human Research Ethics Committee (2021_ETH12472) with informed consent from all participants. Eligible individuals were assigned female at birth, aged over 18 years, presenting with clinical signs or symptoms of endometriosis and undergoing surgical treatment at Prince of Wales Private Hospital, Sydney. Exclusion criteria included pregnancy, suspected malignancy, or insufficient tissue for organoid derivation.

Tissue samples were excised and transported on ice within 4 h in Advanced DMEM/F12 medium (Gibco, #12634028, United States) supplemented with 10% fetal bovine serum (Scientifix, #FBSFR-SOOJF, Australia) and 1% penicillin-streptomycin (Gibco, #15140122, United States). Samples were cryopreserved in RPMI (Gibco, #11875093, United States) containing 40% fetal bovine serum and 10% dimethyl sulfoxide (DMSO; Sigma-Aldrich, #D2650, United States) at −80 °C until processing.

Upon thawing, tissues were minced into ∼5 mm^3^ pieces, rinsed in Ca^2+^/Mg^2+^-free PBS (Gibco, #14190144, United States), and enzymatically digested with 20 μg/mL collagenase IV (Gibco, #17104019, United States) in Advanced DMEM/F12 at 37 °C with agitation (180 rpm) for up to 3 h. Mechanical dissociation was performed every 20 min with progressively smaller pipette tips (25 mL–10 mL to 5 mL). Digestion was halted by adding twice the digestion volume of medium, and large debris was removed by filtering through a 100 µm nylon filter (Corning, #352360, United States). The cell suspension was centrifuged at 220 *g* for 5 min at 4 °C, and the pellet resuspended in 70% growth-factor reduced Matrigel (Corning, #356231, United States).

Triplicate 20 µL Matrigel domes were plated per well in pre-warmed 24-well tissue culture plates (Corning, #3524, United States) and incubated at 37 °C for 15 min to solidify. Wells were overlaid with 500 µL of pre-warmed organoid culture medium supplemented with 1% antibiotic-antimycotic solution (Sigma-Aldrich, #A5955, United States), as previously described (Boretto et al., 2019). Organoids were maintained at 37 °C with 5% CO_2_, with medium changed every two to 3 days depending on growth. Upon reaching approximately 80% confluence, organoids were mechanically passaged by ice-cold medium disruption, centrifugation at 200 *g* for 5 min at 4 °C, and replating in a 1:4 dilution of 70% Matrigel domes as previously described. Passaged cultures received 10 µM Y-27632 ROCK inhibitor (STEMCELL Technologies, #72304, Canada) in organoid medium for enhanced viability.

For histological processing, organoids were collected by centrifugation, embedded in warmed HistoGel (Epredia, #22–110–678, United States), fixed in 4% formalin for 1 h, then transferred to 70% ethanol. HistoGel domes were embedded in paraffin and sectioned at 4 µm for downstream staining.

### 2.6 Functional assays in organoid models

Organoids were cultured to confluence in at least 12 replicate 20 µL Matrigel domes. For passaging, organoids were collected in ice-cold medium and mechanically disrupted to generate a single-cell suspension. Samples were centrifuged at 200 *g* for 5 min at 4 °C to pellet cells. Media was aspirated, and pellets resuspended in 280 µL of 70% Matrigel. Five-microliter domes were plated centrally in pre-warmed 96-well plates and incubated at 37 °C for Matrigel to solidify. Each well received 100 µL of pre-warmed organoid medium containing 10 µM Y-27632 ROCK inhibitor for the first 2 days (STEMCELL Technologies, #72304, Canada). Organoids were incubated for 5 days with standard organoid media changes every other day.

At day 5, drug serial dilutions ranging from 100 μM to 1.56 µM were prepared in standard organoid media. Old media was aspirated, and 100 µL of pre-warmed drug-containing medium was gently added atop the domes in quintuplicate wells. Vehicle controls contained 0.1% DMSO, and positive controls contained 25% DMSO to induce cytotoxicity. Plates were imaged every 4 h for 120 h using the IncuCyte system. Media containing drug concentrations were refreshed at 72 h.

At 120 h, 100 µL of CellTiter-Glo 3D reagent (Promega) was added per well. Matrigel domes were mechanically disrupted, and contents mixed on a plate shaker at 900 rpm for 5 min. After a 25-min incubation at room temperature, luminescence was measured using a Varioskan plate reader (ThermoFisher).

Organoid area was quantified via IncuCyte software with segmentation parameters set to a radius of 200 μm, sensitivity of 30, and edge sensitivity of 50. A minimum area threshold of 1,000 μm^2^ was applied. Wells lacking visible organoids were excluded. Area metrics were normalized to baseline (time zero), and percentage change from baseline was calculated and averaged per timepoint.

### 2.7 Immunohistochemical analysis

Tissue microarrays (TMAs) were constructed as previously described (manuscript under review). The TMAs include 179 tissues, comprising 100 lesions, 56 regions of tissue adjacent to lesions, and 23 matched eutopic endometrium samples. Lesion subtypes included superficial peritoneal endometriosis (SUP, n = 11), deep infiltrating endometriosis (DIE, n = 22), and endometrioma with concurrent DIE (OMA, n = 11). Each tissue region was represented in triplicate, or in duplicate where insufficient material was available. Paraffin-embedded blocks were sectioned at 4 µm and mounted onto SuperFrost Ultra Plus glass slides (Roth). Embedded 12Z spheroids and organoid models were sectioned and mounted similarly. Immunohistochemical staining was performed using a Leica BOND RX automated stainer with the Bond Polymer Refine Detection system (Leica Biosystems).

Slides were baked at 60 °C for 30 min, dewaxed with Bond Dewax solution at 72 °C for 30 s, and subjected to heat-induced epitope retrieval in EDTA buffer (pH 9.0) at 100 °C for 20 min. Endogenous peroxidase activity was quenched by incubation with peroxide block at room temperature for 5 min. Sections were incubated with mouse anti-ROR1 monoclonal antibody (clone 6D4, 0.74 μg/mL; diluted 1:9,000) for 30 min at room temperature, followed by polymer-based secondary detection and chromogenic development using DAB for 10 min. Slides were counterstained with hematoxylin for 5 min, dehydrated through graded ethanol series, cleared in xylene, and coverslipped with Pertex. Antibody dilution was optimized prior to this study using control tissues with known positive and negative expression, as defined by the Human Protein Atlas (Uhlén et al., 2015).

### 2.8 Microscopy and scoring

Slides were imaged in brightfield on Olympus VS200 at ×40 objective. Digital TMA slides were de-arrayed in QuPath and scored blindly by two independent scorers (BG, MH). An additional scorer (CL) evaluated the original physical slides to complement the digital scoring. Each core was scored for maximum staining intensity on an ordinal scale: 0 (absent), 1 (low), 2 (moderate), 3 (high), from which an H-score (0–300) was calculated using the standard formula (Mccarty et al., 1986):

$$H-score=\left({0\times P}_{0}\right)+\left({1\times P}_{1}\right)+\left({2\times P}_{2}\right)+\left({3\times P}_{3}\right)$$

All cells were scored regardless of phenotype. Where discordant scores were obtained, a fourth scorer (KG) was used to achieve consensus.

The maximum staining score of all replicate cores of disease was taken, informed by the biological absence in most healthy adult tissues. For those patients with multiple regions of disease captured, the median of the lesion-level maximum was taken, with values rounded to the nearest whole number. Low expression was classified as a score of 0–1, while high expression was classified as a score of 2–3. These cut-off points were informed by the distribution of data.

### 2.9 Analysis

Comparisons of transcript and protein expression across lesion types were performed using linear mixed-effects models using the lme4 package (v1.1.37) in R (v4.3.1), with patient ID included as a random effect to account for repeated measures. For transcript analysis, tissue type, stage, and menstrual phase included as fixed effects. Bayesian inference was applied via the parameters package, reporting posterior median estimates and 95% highest posterior density (HPD) intervals for group contrasts. Protein expression (immunohistochemistry H-scores) was analyzed analogously with tissue type as a fixed effect and patient ID as a random effect; posterior median contrasts with 95% HPD intervals were reported for all comparisons. Pairwise comparisons were conducted using emmeans (v1.8.6) with bootstrap resampling to estimate uncertainty intervals.

Associations between dichotomized biomarker intensity and patient characteristics (phenotype, stage, hormonal treatment, surgical history) were assessed using Pearson’s chi-square test in SPSS (v28.0), with Fisher’s exact test applied when more than 20% of expected counts were less than 5. Changes in organoid size were calculated relative to baseline (untreated day 5) measurements. Dose-response curves from cell viability and organoid assays were plotted and analyzed using nonlinear regression in GraphPad Prism (v10.2) to estimate IC_50_ values. Statistical significance was considered at p < 0.05.

## 3 Results

### 3.1 ROR1 is transcriptionally upregulated in endometriosis lesions independent of menstrual phase or stage

**FIGURE 1:**
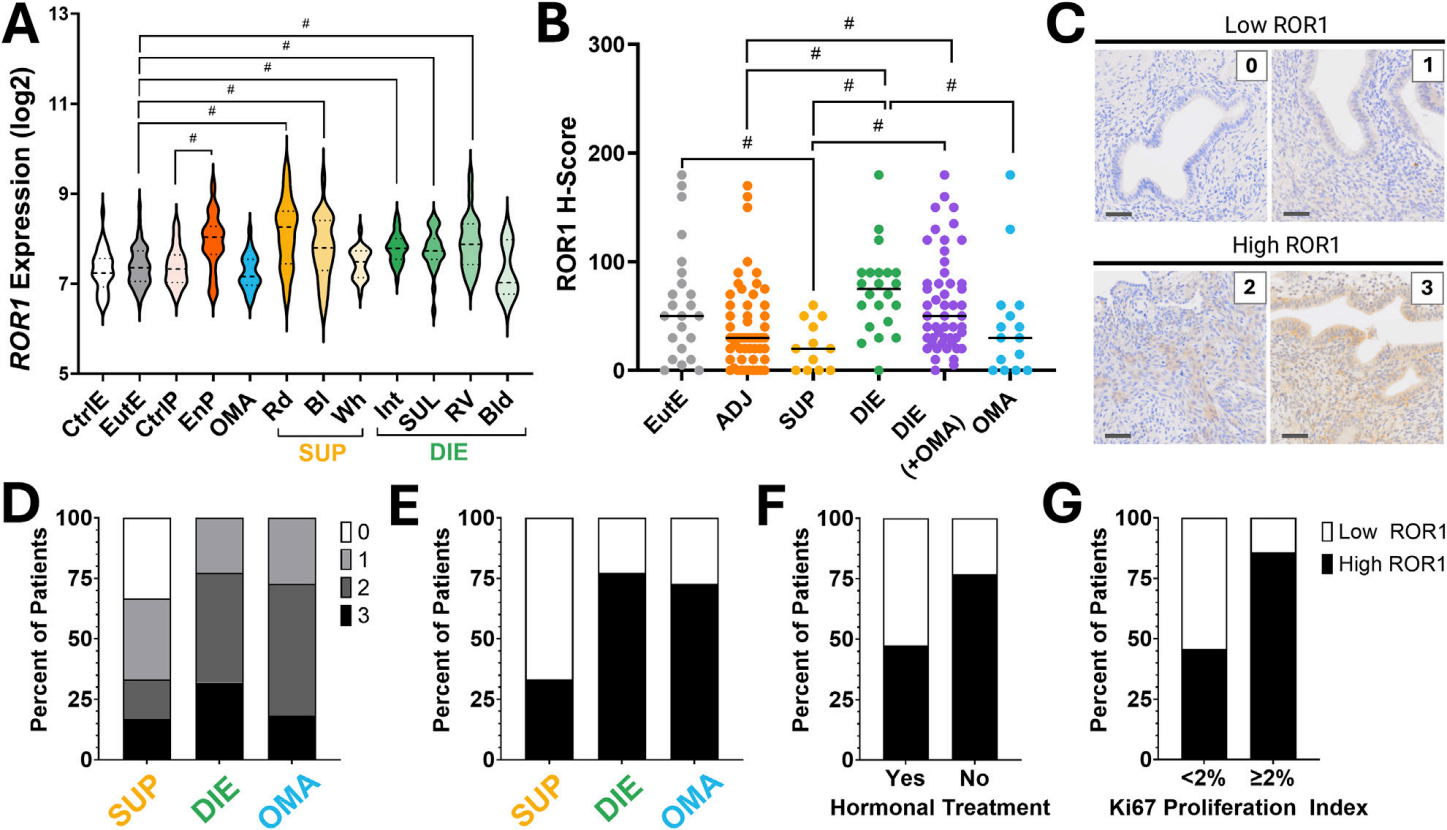
ROR1 Expression from whole tissue endometriosis samples. **(A)** *ROR1* transcript levels in GSE141549 by tissue type and lesion location. Deep infiltrating endometriosis (DIE: intestinal [Int], rectovaginal [RV], sacrouterine ligament [SUL], bladder [Bld]) shown in green; superficial peritoneal endometriosis (SUP: red [Rd], black [Bl], white [Wh]) in yellow; ovarian endometrioma (OMA) in blue; peritoneal tissue adjacent to lesions (EnP) in dark orange; control peritoneum (CtrlP) in light orange; control endometrium (CtrlE) in white; eutopic endometrium (EuE) in grey. **(B)** ROR1 histochemistry score (H-score) for each lesion captured in the tissue microarray by tissue type. Linear mixed-effects models were used to account for multiple lesions from the same patient; line indicates median by phenotype. **(C)** Representative ROR1 immunohistochemistry intensity (0,1,2,3). Scoring thresholds were defined as low expression (0, 1) and high expression (2, 3). **(D–F)** Patient-level ROR1 expression assigned as “low” (H-score 0–1) or “high” (H-score 2–3). For patients with multiple lesion cores, the median of the lesion-level maximum scores was used, rounded to the nearest whole number. **(D)** Patient ROR1 intensity by phenotype. **(E)** Patient ROR1 high or low expression by phenotype. **(F)** Patient ROR1 high or low expression by hormonal treatment. **(G)** Patient ROR1 high or low expression by Ki67 proliferation index. Significance defined as 95% HPD intervals excluding 0 and indicated by is indicated by #. Scale bar = 50 µm.

Analysis of a publicly available microarray dataset comprising 408 samples from 115 patients and 53 controls revealed that ROR1 transcript expression was significantly elevated in deep infiltrating endometriosis (DIE) lesions, including intestine (Estimate −0.511, 95% HPD –0.740 to −0.275), rectovaginal (−0.632, −0.883 to −0.375), and uterosacral ligament sites (−0.445, −0.670 to −0.178), as well as in superficial peritoneal endometriosis (SUP) red (−0.863, −1.112 to −0.634) and black lesions (−0.481, −0.743 to −0.259), compared to control endometrium (Figure 1A; Supplementary Table 1). In contrast, no significant differences were observed in ovarian endometrioma (0.052, −0.170–0.314), SUP white lesions (−0.219, −0.474 to 0.034), DIE bladder lesions (0.064, −0.510–0.619), or eutopic endometrium (−0.106, −0.270 to 0.104). Peritoneal tissue adjacent to endometriotic lesions also showed significantly higher ROR1 expression relative to control peritoneum (−0.557, −0.817 to −0.305). Expression did not vary with menstrual cycle phase or disease stage (Supplementary Table 2).

### 3.2 Elevated ROR1 protein correlates with deep infiltrating endometriosis, lack of hormone treatment, and increased cellular proliferation

ROR1 protein expression, assessed by H-score, varied across tissue types (Figure 1B; Supplementary Table 3). Both deep infiltrating endometriosis (DIE) lesions and DIE with concurrent ovarian endometrioma (DIE + OMA) exhibited higher ROR1 levels than adjacent peritoneal tissue. DIE alone and DIE + OMA were also higher than superficial endometriosis (SUP), while superficial lesions showed lower expression than eutopic endometrium. No significant differences were observed between DIE alone and DIE + OMA, indicating that ROR1 is most elevated in DIE lesions and lowest in SUP.

At the patient level, median ROR1 scores across all lesions were dichotomized as low (0–1) versus high (2–3). High ROR1 was significantly associated with lack of hormonal treatment and increased Ki67, but not with progesterone receptor status, surgical history, disease stage, Endometriosis Fertility Index, infertility, pain scores, family history, or co-diagnoses (Supplementary Table 3; Figures 1E,F). A secondary analysis using absent (0) versus present (1–3) median scores revealed significant associations with disease stage and dyspareunia (p = 0.001 and 0.034, respectively). Multiregional immunohistochemistry revealed intrapatient heterogeneity, with variable expression between lesions and between lesions and adjacent histologically normal tissue. Positivity was detected in stromal and epithelial compartments in different lesions, reflecting a complex and heterogeneous pattern (Supplementary Figure 1).”

### 3.3 Computational chemistry identifies candidate ROR1-Binding agents with consumer and clinician input

**FIGURE 2:**
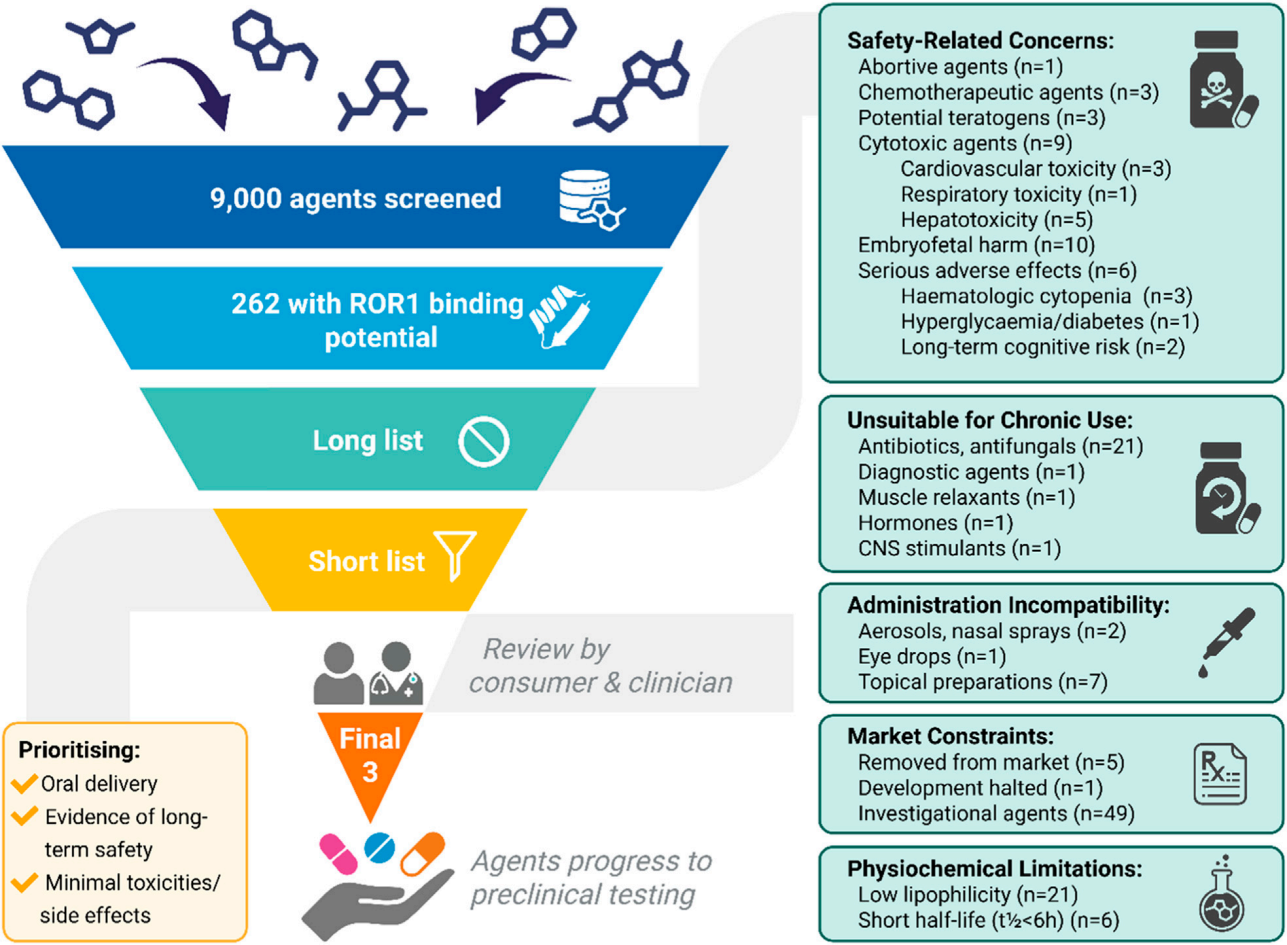
Drug Repurposing Workflow for Computational Prioritization of Candidate Compounds with Predicted ROR1 Binding Potential. Approximately 9,000 compounds were screened *in silico* with the BLAZE™ platform to identify 262 candidates with predicted ROR1 binding potential. The compound list was filtered by excluding those with safety concerns, unsuitability for chronic use, incompatible administration routes, market constraints, and physicochemical limitations. A multidisciplinary panel including a clinician and consumer advocate then prioritized the shortlist based on oral delivery, long-term safety, and minimal toxicity. Three lead compounds were selected for further *in vitro* evaluation. Figure created in BioRender. Abbott, J. (2025) https://BioRender.com/6x4s87e.

Of approximately 9,000 compounds screened using the BLAZE™ platform, 262 (2.9%) demonstrated predicted affinity for the ROR1 binding site between the ROR1-Kringle domain and the antibody heavy chain variable domain. Following initial *in silico* filtering, compounds were excluded primarily due to market availability constraints (n = 55), safety concerns (n = 41), pharmacokinetic or physicochemical limitations (n = 27), unsuitability for long-term or reproductive-age use (n = 26), and incompatible routes of administration (n = 10) (Figure 2).

A shortlist of six candidate compounds was then independently reviewed by a multidisciplinary panel, including a consumer advocate and a clinician, to prioritize compounds based on safety, acceptability, and relevance for patients with endometriosis (Table 1). Consumer input emphasized potential nutritional deficiencies and anticholinergic effects with pirenzepine, whereas clinical feedback suggested no major barriers to its use in patients with endometriosis. For lercanidipine and propranolol, low blood pressure was raised as a consumer concern, while clinical feedback stressed caution in young women with endometriosis due to substantial diurnal blood pressure variation and in specific subsets with combined connective tissue disorders, where restrictions may be required. With ivacaftor, consumer input highlighted hepatotoxicity, gastrointestinal side effects, and a sizeable list of potential drug interactions, and clinical feedback similarly regarded its side effect profile as a key limitation, considering it the least suitable candidate. In contrast, rimegepant drew consumer concern over limited long-term data and possible fertility implications, while clinical feedback viewed it as the most promising option, particularly given its dual utility for migraine. Cabergoline was noted in consumer input for rare dopaminergic behavioral side effects, whereas clinical feedback underscored its familiarity and established role in reproductive endocrinology. This collaborative review refined the shortlist to three lead compounds for subsequent *in vitro* evaluation: cabergoline, pirenzepine, and rimegepant, selected for their tolerability, clinical familiarity, and potential relevance to endometriosis biology.

**TABLE 1:** Pharmacological, safety, and clinical characteristics of repurposed agents against ROR1.

	Pirenzepine	Cabergoline	Rimegepant	Lercanidipine	Propranolol	Ivacaftor
Original Indication	Peptic ulcer	Hyperprolactinemia	Migraine	Hypertension	Hypertension, migraine	Cystic fibrosis
Dose	50 mg PO SID	1 mg PO SID	75 mg PO PRN	10 mg PO SID	40 mg PO BID	150 mg PO BID
logP	0.967	2.579	2.95	6.41	2.58	5.76
Half-life	10 h	63–68 h	11 h	8–10 h	6 h	12 h
C_max_	137 nM	67 nM	1.96 uM (Bhardwaj et al., 2019)	3.30 nM	18 nM	768 nM
Teratogenicity	Not registered	Category B1^s^ Category B^b^	Category B1^a^ Not assigned^b^	Category C^a^	Category C^a^ Category C^b^	Category B3^a^ Not assigned^b^
Mechanism	Antagonist of M1 muscarinic acetylcholine receptor, modulating intracellular K^+^(Carmine and Brogden, 1985) PARP-1 inhibitor (Tropitzsch et al., 2019)	Agonist activity of dopamine and 5-HT receptors, downstream inhibition of intracellular cAMP and intracellular Ca^2+^ (Rains et al., 1995)	CGRP antagonist inhibiting downstream facial vasodilation and nociception (Luo et al., 2012)	Ca^2+^ channel blocker, inhibiting contraction of myocardial smooth muscle cells to cause vasodilation (Meredith, 1999)	β-adrenergic receptor agonist, reducing intracellular cAMP and PKA activity (Sozzani et al., 1992)	Potentiation of CFTR protein channel to increase Cl^−^ transport across the cell membrane (Van Goor et al., 2009)
Side Effects (>5%)^c^	Not registered	Nausea (29%) Headache (26%) Dizziness (17%) Constipation (7%) Asthenia (6%)	N/A	N/A	Sleep disorder (17%) Bronchitis (11%) Peripheral coldness (7%) Agitation (7%) Fatigue (6%)	Headache (24%) Oropharyngeal pain (22%) Rhinitis (20%) Abdominal pain (16%) Diarrhea (13%) Nausea (12%) Dizziness (9%)
Long-Term Use	Continuous use of 12 months well tolerated (Galeone et al., 1983)	Weekly use for 12–24 months well tolerated (Colao et al., 1997) in a prolactinoma cohort. Daily use >6 months associated with increased risk of cardiac valve regurgitation (Trifirò et al., 2012)	Every-other day for 12 weeks or PRN for 12 months well tolerated (Croop et al., 2024)	Daily treatment in diabetic cohort well-tolerated for 12 months (Dalla Vestra et al., 2004)	Twice daily treatment well-tolerated for 8–12 months in migraine cohort (Diamond et al., 1982)	Reported good tolerability in cystic fibrosis cohorts up to 7.9 years (Merlo et al., 2024)
Contraindications^c^	Not registered	uncontrolled hypertension, cardiac vulvar disorders fibrotic disorders	N/A	N/A	Hypoglycaemia, bronchial asthma, cardiac failure bradycardia cocaine	N/A
Drug interactions^c^	Not registered	Dopamine antagonists	CYP3A4 inhibitors CYP3A4 inducers P-gp inhibitors	CYP3A4 inhibitors midazolam other antihypertensives	CYP2D6 inhibitors hypoglycemics, e.g., insulinother antihypertensives	CYP3A inhibitors CYP3A inducers P-gp inhibitors

PO , orally (per os), SID , once daily (semel in die), BID , twice daily (bis in die), PRN , as needed (pro re nata), logP = partition coefficient (measure of lipophilicity), C_max_ = maximum observed plasma concentration, cAMP , cyclic adenosine monophosphate; PKA , protein kinase A, CGRP , calcitonin gene-related peptide; CFTR , cystic fibrosis transmembrane conductance regulator; PARP-1 , poly (ADP-ribose) polymerase 1, N/A = not applicable.

^a^ As defined by TGA Australian Categorisation System for Prescribing Medicines in Pregnancy.

^b^ As defined by the United States Food and Drug Administration (FDA).

^c^ Agent side effects, contraindications and drug interactions were extracted from FDA-approved labeling via DailyMed (National Library of Medicine (Us), 2025).

### 3.4 Rimegepant exhibits cytotoxicity in 2D endometriosis cell line models

**FIGURE 3:**
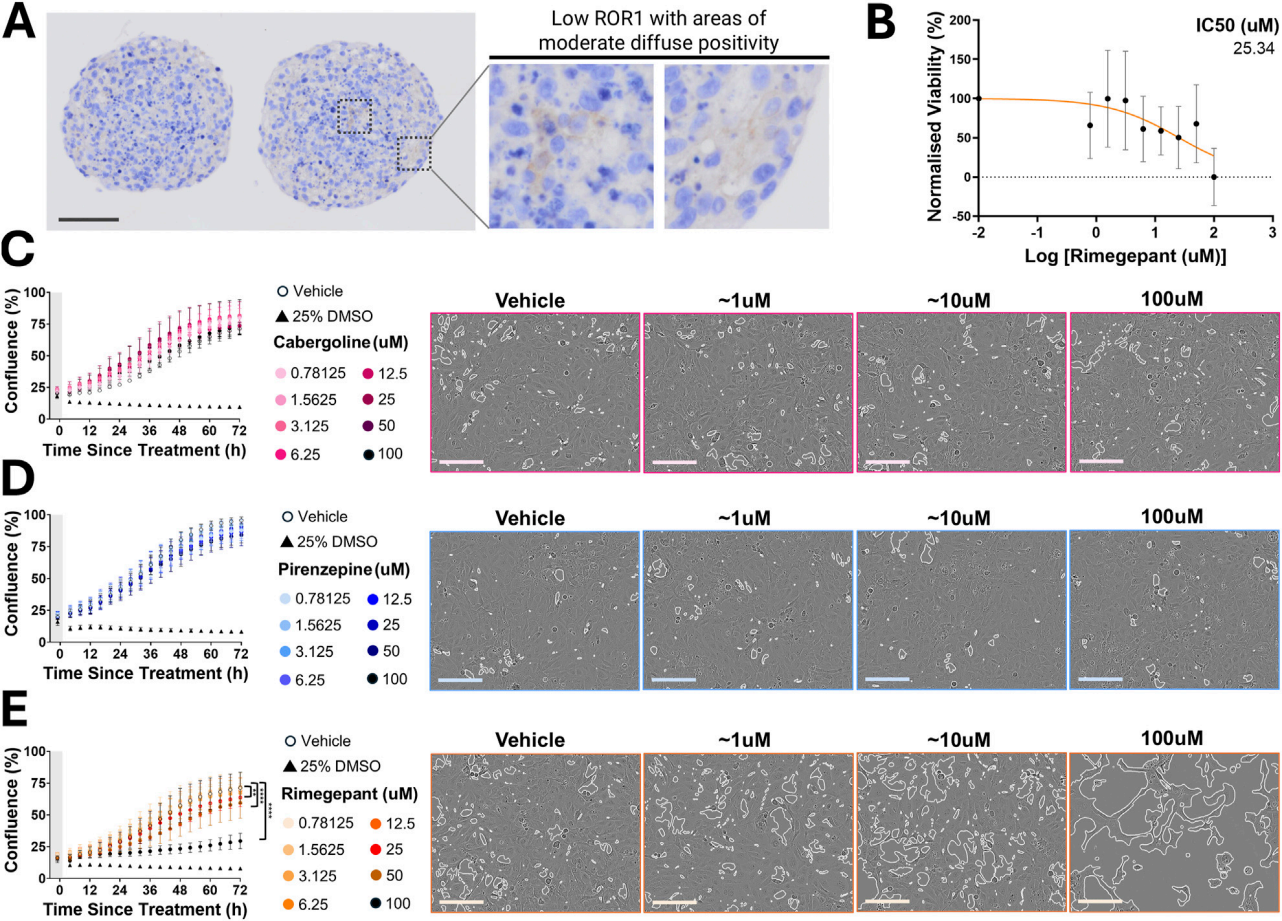
**(A)** ROR1 Expression and Drug Sensitivity of Repurposed Agents in 12Z Endometriotic Epithelial Cell Line. Immunohistochemistry of 12Z spheroids showing low-to-moderate ROR1 expression. Insets show high-magnification areas. Scale bar: 100 µm. **(B)** Estimated IC_50_ for 12Z cells treated with rimegepant in human plasma-like medium (HPLM). **(C–E)** Mean confluence of 12Z cells over 72 h in HPLM following treatment with serial dilutions of cabergoline **(C)**, pirenzepine **(D)**, or rimegepant **(E)**, with representative IncuCyte images at 72 h for vehicle and three concentrations (∼1, ∼10, 100 µM). Cell area was quantified by perimeter detection, with white outline demonstrating areas lacking cells. Scale bars: 200 µm. Grey shading indicates treatment period. Data in **(C-E)** are mean ± SEM; all experiments represent *n* = 3. Significance is indicated by asterisks (*p < 0.05, **p < 0.01, ***p < 0.001).

To evaluate the effects of repurposed agents on endometriosis cell growth under physiologically relevant conditions, functional assays were performed in human plasma-like medium (HPLM). Treatment with cabergoline or pirenzepine had no significant impact on cell viability or proliferation at any tested concentration in HPLM (Figure 3) or in standard DMEM/F12 medium (Supplementary Figure X). In contrast, rimegepant significantly reduced cell viability at 25 μM, 50 μM, and 100 µM in HPLM (Figure 3E), with an estimated IC_50_ of 25.34 µM (Figure 3C).

### 3.5 Rimegepant inhibits growth and viability of Patient-Derived Endometriosis Organoids, revealing interpatient heterogeneity

**FIGURE 4:**
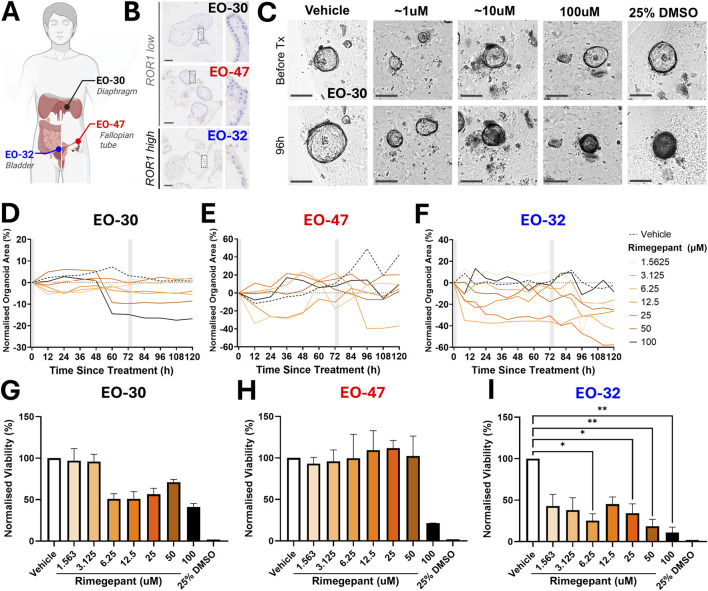
ROR1 Expression and Rimegepant Sensitivity in Patient-Derived Endometriosis Organoids. **(A)** Anatomical location of origin for each endometriosis organoid model: EO-32, intramural bladder endometriosis; EO-30, deep infiltrating endometriosis of the diaphragm; EO-47, deep infiltrating endometriosis of the fallopian tube serosa. **(B)** Representative immunohistochemistry (IHC) images of each organoid model showing low ROR1 protein expression (EO-30, EO-47) and moderate-high ROR1 expression (EO-32). Scale bar: 100 µm. Insets show high-magnification areas. **(C)** Representative brightfield images of EO-30 organoids from IncuCyte either 1 h before treatment or after secondary treatment with agent (96 h treatment; representative of the full 120 h assay), with ∼1 μM, ∼10 μM, 100 μM, and positive control 25% DMSO. No appreciable changes in organoid size, morphology, or color were observed between 96 h and 120 h for this patient. **(D–F)** Mean change in organoid area (%) from baseline (pre-treatment) across a serial dilution of rimegepant from 1.563 µM to 100 µM. Left to right: EO-30, EO-47, EO-32. Data represent the mean of replicate wells per timepoint and treatment, expressed relative to baseline (day 5, pre-treatment). Grey shading indicates treatment period. Error bars omitted for clarity, examples can be seen in Supplementary Figure 3. **(G–I)** Normalized organoid viability following rimegepant serial dilution treatment, relative to vehicle (100%) and 25% DMSO positive control (0%). Data shown as mean ± SEM. From left to right: EO-30, EO-47, EO-32. *p < 0.05, **p < 0.01. Figure partially created in BioRender. Abbott, J. (2025) https://BioRender.com/6x4s87e.

Organoid models were established from deep infiltrating endometriosis tissues obtained from three patients: bladder wall (EO-32), diaphragm (EO-30), and fallopian tube serosa (EO-47) (Figure 4A). Immunohistochemistry confirmed high ROR1 expression in EO-32, with lower expression in EO-30 and EO-47 (Figure 4B).

Rimegepant treatment revealed interpatient heterogeneity. Growth analysis showed that EO-30 organoids markedly decreased in size at concentrations >3.125 µM (Figure 4D), EO-32 at concentrations ≥3.125 µM (Figure 4E), while EO-47 demonstrated a more mixed response (Figure 4F). EO-30 is shown as a representative example, where organoids exposed to ≥12.5 µM not only shrank but also darkened, reflecting cytotoxic changes that complement the measured decrease in size (Figure 4C). Viability assays supported these findings. EO-32 showed an early, though non-significant, decrease in cell viability at 1.563 µM, with a significant reduction at 6.25 µM (Figure 4I). EO-30 displayed ∼50% cell viability at 6.25 µM that persisted across higher concentrations, although changes were not statistically significant (Figure 4G). EO-47 maintained viability until 100 μM, where cell viability dropped to ∼20% without reaching significance (Figure 4H).

## 4 Discussion

Despite a growing understanding of endometriosis pathophysiology, effective management of disease remain a challenge for many patients due to heterogeneity and the limited therapeutic options currently available. Through the integration of molecular profiling, computational drug repurposing, and patient-derived functional assays to evaluate candidate therapies in a clinically relevant context we have demonstrated the power of a multimodal approach to address this challenge. By utilizing both 2D and 3D epithelial models alongside patient-informed prioritization, we provide proof-of-concept that repurposed agents can be systematically assessed for feasibility, efficacy, and translational potential in endometriosis, highlighting both the promise of novel molecular candidates such as ROR1 and the broader value of repurposing to accelerate effective, well-tolerated therapies.

ROR1 was selected as a candidate target based on its restricted expression in healthy adult tissues and its emerging role in precision oncology, making it an attractive molecule for therapeutic intervention (Balakrishnan et al., 2017; John and Ford, 2022). Analysis of endometriotic lesions revealed a significant association between high ROR1 expression and elevated Ki67 proliferation indices, a marker previously linked to disease recurrence (Yalcin et al., 2017). High ROR1 expression was also more frequently observed in patients not receiving hormonal treatment at the time of surgery, suggesting potential relevance for individuals in whom hormonal therapies are contraindicated, ineffective, or undesired. *ROR1* was transcriptionally upregulated in both endometriotic lesions and macroscopically normal-appearing peritoneum from patients with endometriosis, while protein expression in adjacent tissue cores was comparable to or lower than in lesions, reflecting heterogeneity within the local microenvironment (Supplementary Figure 1). This variability may relate to occult endometriosis in macroscopically normal peritoneum (Gubbels et al., 2020), or to ROR1’s involvement in biological pathways relevant to lesion pathophysiology, including inflammation (Barnicle et al., 2017; Nema et al., 2021), fibrosis (D’alessandro et al., 2021; Zhang et al., 2025; Chavkin et al., 2021), and neural remodeling (Takahashi et al., 2022; Paganoni et al., 2010). While these findings provided the rationale for exploring ROR1-targeted therapies, they also raise questions about potential off-target effects and safety considerations in normal tissues, highlighting the importance of careful preclinical evaluation and repurposing strategies.

Initial functional evaluation was conducted using the 2D endometriosis cell line 12Z to screen three candidate repurposed agents, rimegepant, cabergoline, and pirenzepine, identified through the computational ROR1-binding prioritization pipeline and selected through consumer and clinician input. Incorporating perspectives from those with lived experience ensured that prioritization aligned with patient-defined values of safety, tolerability, and acceptability, while clinical feedback helped anticipate pharmacological considerations relevant to endometriosis comorbidities. Of the three agents, only rimegepant induced a significant change in proliferation (Figures 3D–F), justifying further evaluation of rimegepant in patient-derived 3D organoid models. Rimegepant is a clinically approved small-molecule antagonist of calcitonin gene-related peptide (CGRP), prescribed for acute migraine at a 75 mg oral dose, producing a producing a C_max_ of approximately 1.96 µM in clinical pharmacokinetic studies, a concentration established for CGRP inhibition (Bhardwaj et al., 2019). CGRP is a neuropeptide with well-established roles in pain transmission, vasodilation, and neurogenic inflammation (Iyengar et al., 2017; Urits et al., 2020; Sohn et al., 2020), and emerging evidence also implicates CGRP in endometriosis lesion biology and pain signaling (Fattori et al., 2024; Raffaelli et al., 2021; Chaichian et al., 2024). Taken together, these observations support the functional relevance of rimegepant in endometriosis and justify further evaluation in human-derived organoid models.

In our organoid models, some patient-derived lines responded to concentrations moderately above this level, whereas EO-47 did not show significant cytotoxicity at the clinically meaningful concentrations tested, indicating a level of patient heterogeneity. While direct comparison of C_max_ and *in vitro* potency has limitations, repeat or higher dosing of rimegepant has been shown to be safe, with administration up to 600 mg/day for 14 days or single doses up to 1,500 mg achieving plasma exposures exceeding those that elicited effects in responsive organoid (22.86uM and 15.02uM, respectively) (Bertz et al., 2023). Rimegepant also benefits from a well-characterized safety profile with minimal side effects, which may circumvent concerns regarding off-target effects in normal tissues, including those expressing ROR1.

Although the observed effects in organoids may not be purely ROR1-dependent, they demonstrate the utility of the repurposing pipeline in identifying candidate agents with translational potential. These findings complement prior preclinical studies in animal models, which showed that rimegepant reduced lesion size and pain behaviors (Fattori et al., 2024), supporting the relevance of CGRP in endometriosis (Chaichian et al., 2024; Raffaelli et al., 2021). Importantly, the present study provides the first evidence in human-derived endometriosis models that rimegepant can influence cell growth and viability. To further evaluate ROR1 as a therapeutic target, future studies could assess its function in endometriosis-like lesions *in vivo*, providing an opportunity to validate ROR1’s role in lesion initiation, progression and recurrence. Overall, these results illustrate how molecularly guided drug repurposing, integrated with patient-derived functional models can generate human-relevant hypotheses, accelerate candidate prioritization, and reduce reliance on early-stage preclinical safety testing in animals.

In 3D organoids, rimegepant-treated EO-47 exhibited limited cytotoxicity, but growth was effectively halted at concentrations above 3.125 µM, whereas rimegepant-treated EO-30 exposed to 10 µM showed marked decreases in size and darkening consistent with cell death (Figure 4C). These observations highlight that organoid morphology alone does not always directly reflect cellular viability or treatment effect. In endometriosis, symptom severity, particularly pain, often correlates poorly with lesion burden, meaning that cytotoxicity *in vitro* may not translate to clinically meaningful outcomes. This underscores the importance of the multiple complementary metrics recorded in this study, including organoid size, growth dynamics over time, and viability measures, which together provide a more holistic view of drug response.

A notable strength of this study was the early integration of lived-experience perspectives and clinical feedback into the drug selection process, ensuring that experimental priorities reflected patient and clinician-defined needs. In endometriosis, fewer than a quarter of patients are satisfied with current treatments, a dissatisfaction often linked to systemic barriers and a lack of holistic care (Evans et al., 2021). Over a third of young adults with endometriosis identify a critical unmet need for therapies that are both more effective and have fewer side effects (Taffs et al., 2024), and identification of optimal treatment strategies remains the highest research priority among those living with the condition (Armour et al., 2023). These patient-driven priorities underscore the importance of incorporating stakeholder perspectives into translational pipelines. Other drug repurposing initiatives, such as those within the SIMPATHIC consortium for rare neurological and metabolic disorders, have demonstrated that early-stage patient engagement can shape compound prioritization and accelerate clinical translation (Van Karnebeek et al., 2025).

This study has several limitations that should be considered when interpreting the findings. The organoid models were derived exclusively from deep infiltrating endometriosis (DIE) lesions, and therefore the functional findings may not be generalizable to other endometriosis phenotypes. Further, all organoid models were epithelial monocultures, lacking stromal, endothelial, immune, and neuronal cell populations that may influence agent response *in vivo*. For example, co-culture of epithelial organoids with cancer-associated fibroblasts has shown a reduction in drug response compared to monocultures in models of ovarian, pancreatic and colorectal cancers (Ma et al., 2025; Wang et al., 2025; Schuth et al., 2022). In models of endometrium, cocultures of epithelial organoids with endometrial stromal cells have exhibited a lower proliferative index with progestin treatment compared to organoid monoculture (Gnecco et al., 2023). The effect this may have on endometriosis organoids remains theoretical. Immunohistochemistry was scored using whole-core expression metrics (highest intensity/H-score) and did not differentiate between epithelial and stromal compartments. Review of cores (Supplementary Figure 1) indicates variable ROR1 expression across epithelial and stromal cells, and prior research (Yotova et al., 2017) has identified ROR1 upregulation in stromal cells. Such compartment-specific expression may have related or independent functional consequences that could influence lesion responsiveness to treatment. These factors limitations of current monoculture organoid models of endometriosis and underscore the need for future studies incorporating additional cell types to create more complex co-culture systems for drug investigational studies.

Cultures were maintained in media containing 17β-estradiol (E_2_) but no progesterone, which does not replicate the cyclical hormonal milieu. Given that serum CGRP shows cyclical expression across the menstrual cycle that is attenuated with use of hormonal contraceptives (Raffaelli et al., 2021; Raffaelli et al., 2023), rimegepant activity observed may not reflect responses under luteal or contraceptive conditions. Lesion-specific hormone receptor heterogeneity may add a further layer of complexity (Colgrave et al., 2024). Rimegepant emerged in the drug repurposing pipeline due to potential interactions with ROR1, which appears stably expressed across the cycle in endometriosis lesions, while systemic CGRP levels fluctuate. Whether either factor or their combination modulates rimegepant efficacy across hormonal fluctuations remains unknown. Systematic evaluation across hormone contexts, including progesterone or progestin co-treatment, could clarify phase or regimen-dependent efficacy.

This study underscores the potential of drug repurposing as a translational strategy in endometriosis. By integrating molecular profiling, functional organoid assays, and patient-informed prioritization, this pipeline provides a framework to identify candidate therapies with both biological plausibility and practical feasibility. Future studies should expand to include additional lesion phenotypes, co-culture models incorporating stromal and immune components, and longitudinal evaluation of organoid dynamics to better predict clinical response. Such approaches will help bridge preclinical observations with patient-centered outcomes, informing the design of precision-guided clinical trials and ultimately broadening the therapeutic arsenal for endometriosis.

## Data Availability

The datasets presented in this study can be found in online repositories. The names of the repository/repositories and accession number(s) can be found below: https://www.ncbi.nlm.nih.gov/geo/, GSE141549 https://www.ncbi.nlm.nih.gov/geo/, GSE148473.
