# Supplementary material for: An integrated multimodal approach to drug repurposing in endometriosis, using ROR1 as a target
Source: Front Pharmacol. 2025 Dec 3;16:1716062. doi: 10.3389/fphar.2025.1716062 (PMC12709133; doi:10.3389/fphar.2025.1716062)
Supplement: Supplementary file 1 [file Supplementaryfile1.docx]

Supplementary Material

**Supplementary Table 1- Pairwise comparisons of *ROR1* transcript expression (from GSE141549) across endometriosis lesion subtypes and controls**

| **Contrast** | **Estimate** | **95% HPD interval** | **Significant?** |
| --- | --- | --- | --- |
| Control endometrium - Control peritoneum | -0.099 | [-0.337, 0.149] | No |
| Control endometrium - (DIE - bladder) | 0.064 | [-0.510, 0.619] | No |
| Control endometrium - (DIE - intestine) | -0.511 | [-0.740, -0.275] | Yes |
| Control endometrium - (DIE - rectovaginal) | -0.632 | [-0.883, -0.375] | Yes |
| Control endometrium - (DIE - usl) | -0.445 | [-0.670, -0.178] | Yes |
| Control endometrium - Endo peritoneum | -0.662 | [-0.879, -0.441] | Yes |
| Control endometrium - Eutopic endometrium | -0.106 | [-0.270, 0.104] | No |
| Control endometrium - OMA | 0.052 | [-0.170, 0.314] | No |
| Control endometrium - (SUP - black) | -0.481 | [-0.743, -0.259] | Yes |
| Control endometrium - (SUP - red) | -0.863 | [-1.112, -0.634] | Yes |
| Control endometrium - (SUP - white) | -0.219 | [-0.474, 0.034] | No |
| Control peritoneum - (DIE - bladder) | 0.173 | [-0.368, 0.806] | No |
| Control peritoneum - (DIE - intestine) | -0.406 | [-0.687, -0.145] | Yes |
| Control peritoneum - (DIE - rectovaginal) | -0.531 | [-0.789, -0.226] | Yes |
| Control peritoneum - (DIE - usl) | -0.336 | [-0.621, -0.080] | Yes |
| Control peritoneum - Endo peritoneum | -0.557 | [-0.817, -0.305] | Yes |
| Control peritoneum - Eutopic endometrium | 0.002 | [-0.218, 0.224] | No |
| Control peritoneum - OMA | 0.154 | [-0.086, 0.433] | No |
| Control peritoneum - (SUP - black) | -0.376 | [-0.648, -0.114] | Yes |
| Control peritoneum - (SUP - red) | -0.762 | [-1.026, -0.472] | Yes |
| Control peritoneum - (SUP - white) | -0.125 | [-0.390, 0.153] | No |
| (DIE - bladder) - (DIE - intestine) | -0.585 | [-1.154, -0.054] | Yes |
| (DIE - bladder) - (DIE - rectovaginal) | -0.700 | [-1.307, -0.119] | Yes |
| (DIE - bladder) - (DIE - usl) | -0.517 | [-1.074, 0.093] | No |
| (DIE - bladder) - Endo peritoneum | -0.726 | [-1.317, -0.187] | Yes |
| (DIE - bladder) - Eutopic endometrium | -0.174 | [-0.716, 0.384] | No |
| (DIE - bladder) - OMA | -0.027 | [-0.541, 0.623] | No |
| (DIE - bladder) - (SUP - black) | -0.552 | [-1.091, 0.047] | No |
| (DIE - bladder) - (SUP - red) | -0.932 | [-1.473, -0.333] | Yes |
| (DIE - bladder) - (SUP - white) | -0.297 | [-0.882, 0.280] | No |
| (DIE - intestine) - (DIE - rectovaginal) | -0.124 | [-0.395, 0.122] | No |
| (DIE - intestine) - (DIE - usl) | 0.068 | [-0.184, 0.321] | No |
| (DIE - intestine) - Endo peritoneum | -0.149 | [-0.384, 0.064] | No |
| (DIE - intestine) - Eutopic endometrium | 0.407 | [0.206, 0.584] | Yes |
| (DIE - intestine) - OMA | 0.561 | [0.296, 0.794] | Yes |
| (DIE - intestine) - (SUP - black) | 0.026 | [-0.236, 0.261] | No |
| (DIE - intestine) - (SUP - red) | -0.347 | [-0.600, -0.097] | Yes |
| (DIE - intestine) - (SUP - white) | 0.286 | [0.025, 0.533] | Yes |
| (DIE - rectovaginal) - (DIE - usl) | 0.182 | [-0.091, 0.437] | No |
| (DIE - rectovaginal) - Endo peritoneum | -0.025 | [-0.299, 0.209] | No |
| (DIE - rectovaginal) - Eutopic endometrium | 0.528 | [0.287, 0.732] | Yes |
| (DIE - rectovaginal) - OMA | 0.688 | [0.424, 0.987] | Yes |
| (DIE - rectovaginal) - (SUP - black) | 0.151 | [-0.093, 0.412] | No |
| (DIE - rectovaginal) - (SUP - red) | -0.224 | [-0.502, 0.032] | No |
| (DIE - rectovaginal) - (SUP - white) | 0.407 | [0.133, 0.724] | Yes |
| (DIE - usl) - Endo peritoneum | -0.218 | [-0.448, 0.039] | No |
| (DIE - usl) - Eutopic endometrium | 0.338 | [0.116, 0.518] | Yes |
| (DIE - usl) - OMA | 0.494 | [0.247, 0.786] | Yes |
| (DIE - usl) - (SUP - black) | -0.035 | [-0.275, 0.236] | No |
| (DIE - usl) - (SUP - red) | -0.418 | [-0.666, -0.159] | Yes |
| (DIE - usl) - (SUP - white) | 0.223 | [-0.066, 0.491] | No |
| Endo peritoneum - Eutopic endometrium | 0.552 | [0.391, 0.740] | Yes |
| Endo peritoneum - OMA | 0.705 | [0.490, 0.963] | Yes |
| Endo peritoneum - (SUP - black) | 0.175 | [-0.045, 0.435] | No |
| Endo peritoneum - (SUP - red) | -0.204 | [-0.439, 0.035] | No |
| Endo peritoneum - (SUP - white) | 0.435 | [0.205, 0.693] | Yes |
| Eutopic endometrium - OMA | 0.153 | [-0.052, 0.352] | No |
| Eutopic endometrium - (SUP - black) | -0.380 | [-0.612, -0.193] | Yes |
| Eutopic endometrium - (SUP - red) | -0.755 | [-0.975, -0.555] | Yes |
| Eutopic endometrium - (SUP - white) | -0.119 | [-0.331, 0.117] | No |
| OMA - (SUP - black) | -0.533 | [-0.794, -0.306] | Yes |
| OMA - (SUP - red) | -0.906 | [-1.182, -0.649] | Yes |
| OMA - (SUP - white) | -0.277 | [-0.553, -0.015] | Yes |
| (SUP - black) - (SUP - red) | -0.374 | [-0.642, -0.124] | Yes |
| (SUP - black) - (SUP - white) | 0.263 | [0.006, 0.526] | Yes |
| (SUP - red) - (SUP - white) | 0.639 | [0.355, 0.914] | Yes |

*SUP= superficial endometriosis, DIE= deep infiltrating endometriosis, OMA= ovarian endometrioma, endo peritoneum= normal appearing peritoneum from endometriosis patient*

**Supplementary Table 2. Linear mixed-effects model of ROR1 transcript expression.**

Tissue Type, Stage, and Menstrual Phase were included as fixed effects, with Patient ID as a random effect to account for repeated measures. Coefficients represent the estimated difference in log₂ ROR1 expression relative to the reference level for each predictor. P-values test whether each coefficient differs from zero after adjusting for all other variables and accounting for intra-patient correlation. 95% confidence intervals (CI) indicate the uncertainty around each estimate. No significant differences were observed for Stage or Menstrual Phase

| **Predictor** | **Level** | **Coefficient** | **95% CI** | **p-value** |
| --- | --- | --- | --- | --- |
| Tissue Type | DIE – bladder | -0.35 | [-1.48, 0.74] | 0.568 |
| Tissue Type | DIE – intestine | 0.30 | [-0.70, 1.29] | 0.578 |
| Tissue Type | DIE – rectovaginal | 0.45 | [-0.57, 1.45] | 0.398 |
| Tissue Type | DIE – usl | 0.30 | [-0.71, 1.33] | 0.550 |
| Tissue Type | Endo peritoneum | 0.49 | [-0.51, 1.48] | 0.370 |
| Tissue Type | Eutopic endometrium | -0.08 | [-1.02, 0.91] | 0.886 |
| Tissue Type | OMA | -0.23 | [-1.24, 0.76] | 0.668 |
| Tissue Type | SUP – black | 0.29 | [-0.70, 1.32] | 0.580 |
| Tissue Type | SUP – red | 0.70 | [-0.28, 1.70] | 0.190 |
| Tissue Type | SUP – white | 0.06 | [-0.92, 1.06] | 0.934 |
| Stage | I | -0.20 | [-0.70, 0.21] | 0.366 |
| Stage | II | -0.20 | [-0.69, 0.21] | 0.340 |
| Stage | III | -0.35 | [-0.78, 0.06] | 0.096 |
| Stage | IV | -0.12 | [-0.54, 0.29] | 0.524 |
| Menstrual Phase | Medication | -0.40 | [-1.09, 0.20] | 0.224 |
| Menstrual Phase | Menstruation | -0.47 | [-1.16, 0.22] | 0.172 |
| Menstrual Phase | Proliferative | -0.35 | [-1.04, 0.27] | 0.290 |
| Menstrual Phase | Secretory | -0.40 | [-1.09, 0.21] | 0.214 |
| Menstrual Phase | Unknown | -0.32 | [-1.03, 0.34] | 0.366 |

*SUP= superficial endometriosis, DIE= deep infiltrating endometriosis, OMA= ovarian endometrioma, endo peritoneum= normal appearing peritoneum from endometriosis patient. Reference categories: Control endometrium for Tissue Type, Stage 1 for Stage, and Unknown/Not assigned for Menstrual Phase. Coefficients represent the difference relative to the reference.*

**Supplementary Table 3- Pairwise comparisons of ROR1 protein expression (H-score) across endometriosis tissue subtypes**

| **Contrast** | **Estimate** | **95% HPD interval** | **Significant?** |
| --- | --- | --- | --- |
| ADJ - DIE | -31.545 | [-56.700, -11.282] | Yes |
| ADJ - (DIE (+OMA)) | -21.906 | [-37.910, -6.766] | Yes |
| ADJ - EutE | -21.842 | [-41.600, 0.494] | No |
| ADJ - OMA | -4.017 | [-27.560, 19.813] | No |
| ADJ - SUP | 17.669 | [-12.090, 43.647] | No |
| DIE - (DIE (+OMA)) | 9.425 | [-11.780, 31.915] | No |
| DIE - EutE | 9.976 | [-13.240, 37.593] | No |
| DIE - OMA | 28.183 | [2.080, 59.767] | Yes |
| DIE - SUP | 49.217 | [19.540, 83.725] | Yes |
| (DIE (+OMA)) - EutE | 0.153 | [-19.630, 21.596] | No |
| (DIE (+OMA)) - OMA | 18.092 | [-7.880, 40.773] | No |
| (DIE (+OMA)) - SUP | 39.903 | [11.640, 69.491] | Yes |
| EutE - OMA | 18.289 | [-9.880, 44.612] | No |
| EutE - SUP | 38.609 | [6.100, 67.598] | Yes |
| OMA - SUP | 21.260 | [-7.870, 55.692] | No |

*ADJ= adjacent tissue to endometriosis lesion, DIE= deep infiltrating endometriosis, OMA= ovarian endometrioma, EutE= eutopic endometrium, SUP= superficial endometriosis*

**Supplementary Table 3- Statistical associations between clinical characteristics, patient-reported outcomes, and ROR1 expression in endometriosis tissue microarrays**

|  | **Presence/** **Absence by Median** | **Low (01) vs High (23) by Median of Max** |
| --- | --- | --- |
| Phenotype | **<.001** | **0.034** |
| Stage (all) | **0.001** | 0.071 |
| Early vs Late Stage | 0.234 | 0.123 |
| Treatment | 0.461 | **0.014** |
| Surgery Hx Primary vs Recurrent | 1.000 | 0.443 |
| Surgery Hx Primary vs non-primary | 0.712 | 0.634 |
| EFI (low vs high) | 0.249 | 0.322 |
| Infertility | 0.127 | 0.272 |
| Dx Family History | 0.222 | 0.172 |
| Inflammatory Dx | 0.692 | 0.757 |
| Autoimmune Dx | 1.000 | 0.396 |
| Mental Health Dx | 1.000 | 0.903 |
| Gynae Dx | 0.240 | 1.000 |
| Dyspareunia | **0.034** | 0.276 |
| Dyschezia | 1.000 | 0.369 |
| Migraine | 1.000 | 0.586 |
| Fatigue | 1.000 | 1.000 |
| EHP-30 Pain | 1.000 | 0.235 |
| Ki67 High/Low | 0.143 | **0.005** |
| PR any absence | 0.065 | 0.207 |
| PR low/high | 0.142 | 0.056 |
| Recurrence | 1.000 | 0.404 |

*Statistical significance was defined as p < 0.05 and is indicated in* ***bold****.*

**Supplementary Figure 1- Multiregional ROR1 Protein Expression in Endometriosis and Control Tissues**


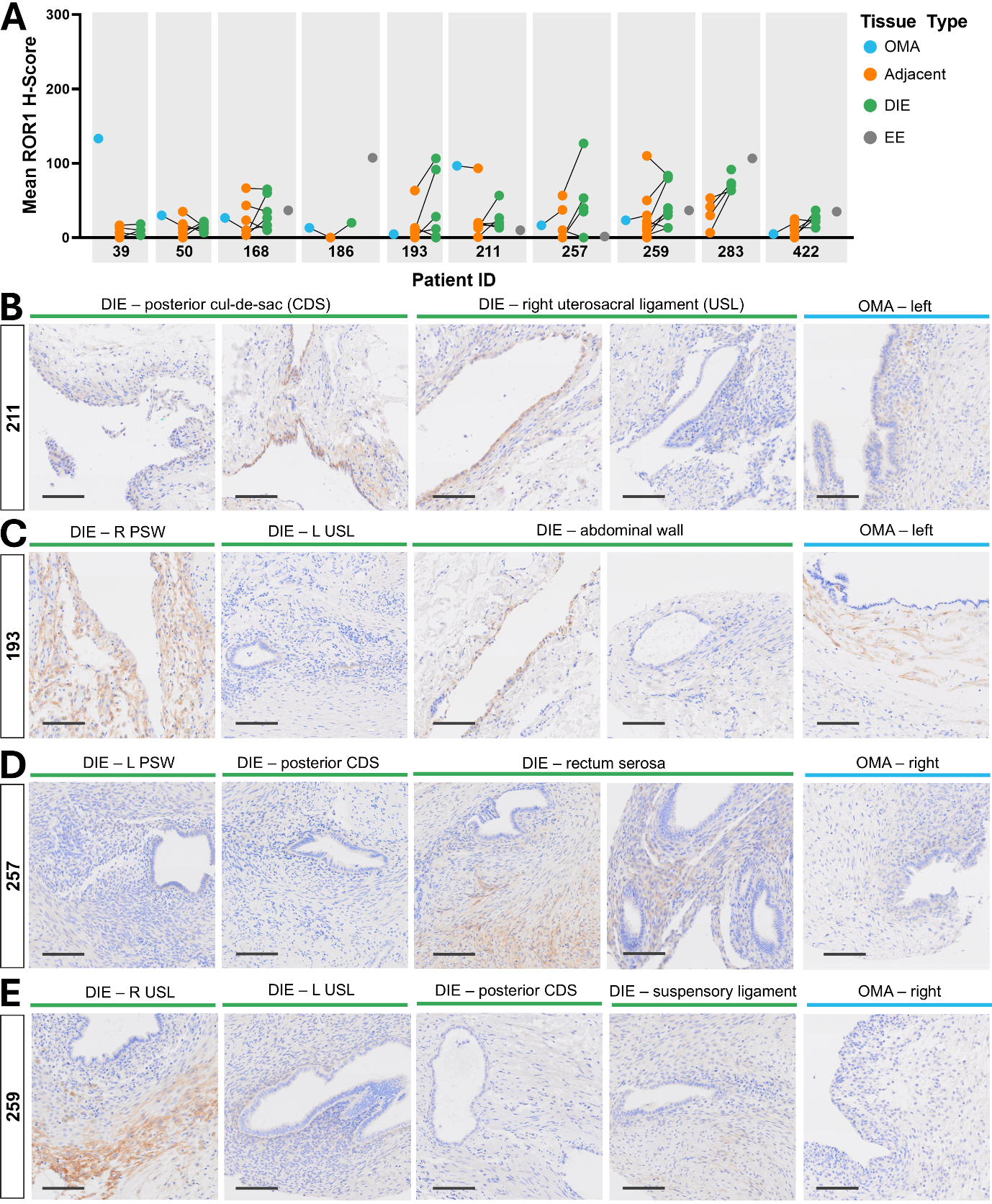


**A)** Multiregional ROR1 immunohistochemistry (IHC) H-scores by disease type: eutopic endometrium (EE), endometrioma (OMA), deep infiltrating endometriosis (DIE), and histologically normal parenchyma adjacent to endometriosis lesions (Adjacent). Each dot represents the mean of technical replicates for that lesion. Lines indicate matched lesion/adjacent region. **B–E)** Representative IHC images of ROR1 expression in tissue microarray cores from indicated anatomical sites. Each row represents samples from a single patient. Labels indicate lesion location. Scale bar = 100 µm. Abbreviations: R, right; L, left; PSW, pelvic side wall; USL, uterosacral ligament; CDS, cul-de-sac; lig., ligament.

**Supplementary Figure 2- Further Validation of ROR1 Expression and Drug Sensitivity of Repurposed Agents in 12Z Endometriotic Epithelial Cells in Alternate Culture Medium**


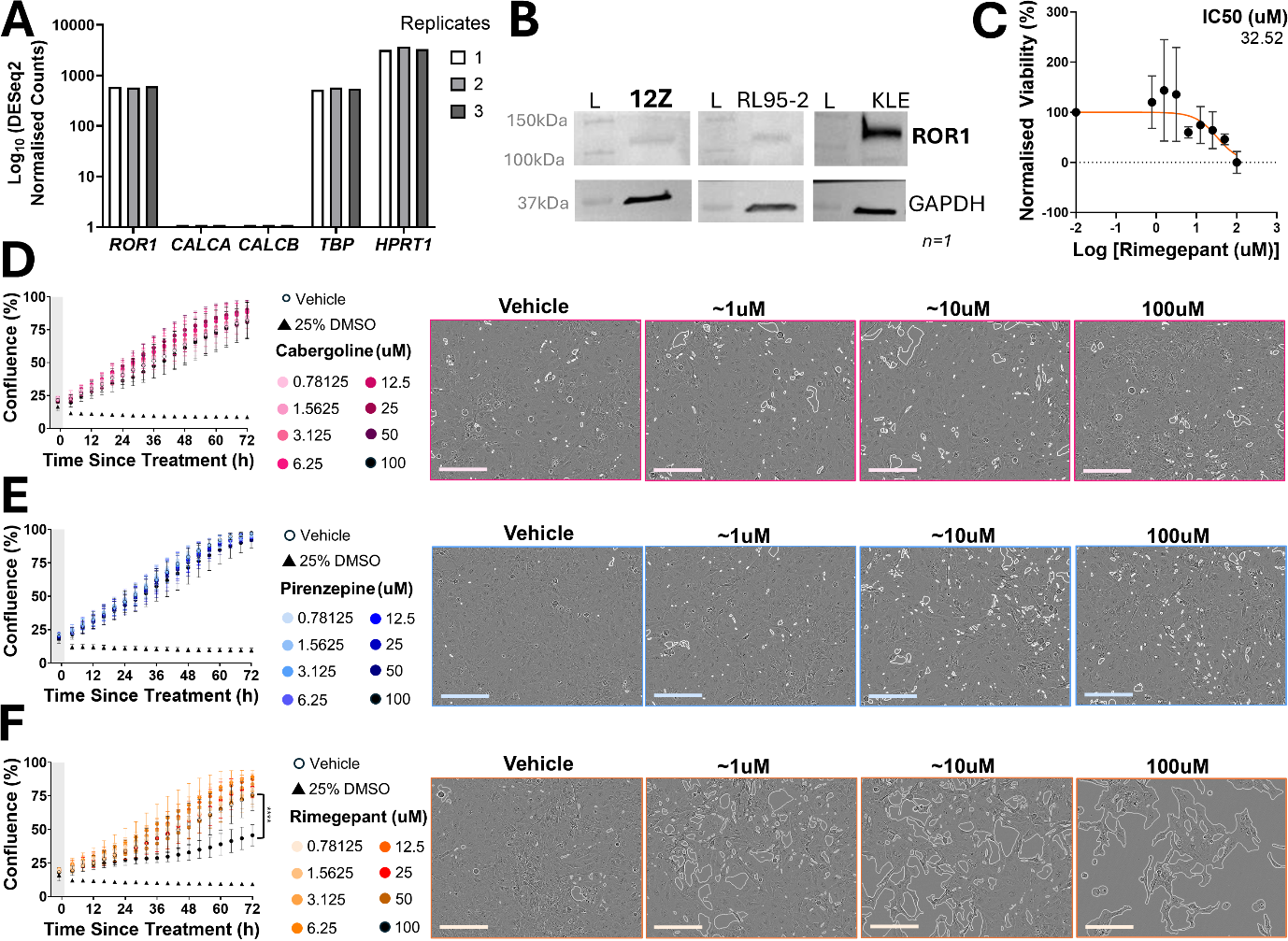


**A)** Normalised expression values for *ROR1,* and CGRP encoding genes *CALCA* and *CALCB* in endometriosis epithelial cell line, 12Z, from GSE148473. Graphed next to reference genes *TBP* and *HPRT* which are moderately expressed. **B)** ROR1 protein (R&D Systems, #AF2000) expression detected by Western blot in 12Z cells and endometrial cancer cell lines RL95-2 and KLE, with GAPDH (Cell signaling technology, #5174) as a loading control. **C)** Estimated IC₅₀ for 12Z cells treated with rimegepant in standard culture medium, complete DMEM/F12. **D)** **E-G)** Mean confluence of 12Z cells over 72 h in complete DMEM/F12 following treatment with serial dilutions of cabergoline (E), pirenzepine (F), or rimegepant (G), with representative images at 72 h for vehicle and three concentrations (~1, ~10, 100 µM). Cell area was quantified by perimeter detection. Scale bars: 200 µm. Grey shading indicates treatment period. Data in C-F are mean ± SEM; all experiments represent *n* = 3 unless otherwise stated. Significance is indicated by asterisks (***p < 0.001).

**Supplementary Figure 3 - Dynamic Growth Responses of Endometriosis Patient-Derived Organoids to Rimegepant Treatment**

**
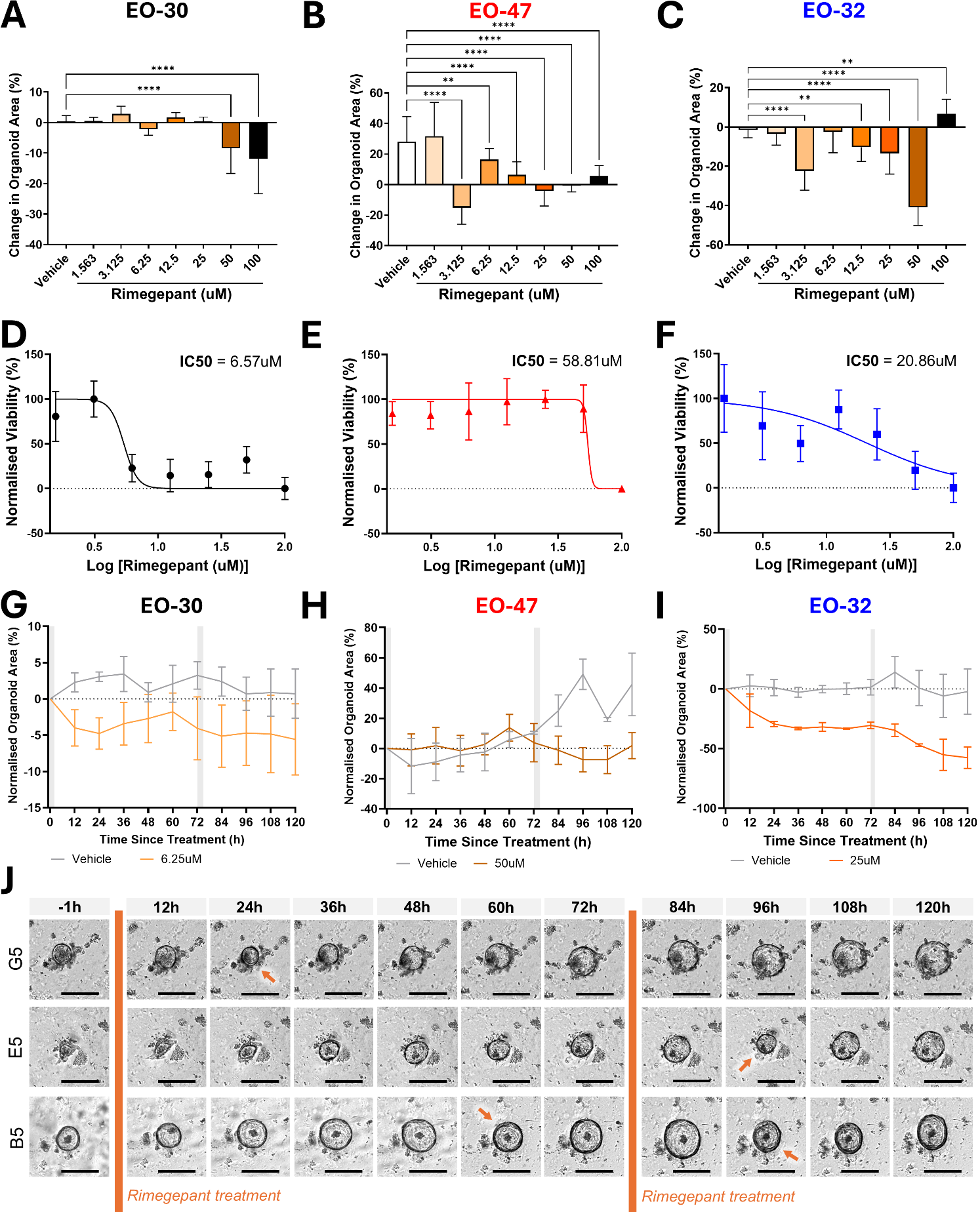
**

**A-C)** Net change in organoid size (% from baseline), calculated as the mean across all timepoints for each condition. Left to right: EO-30, EO-47, EO-32. Statistical comparisons to vehicle were performed using two-way ANOVA. Significance is indicated by asterisks (*p<0.05, **p<0.01, ***p < 0.001)  **D-F)** Estimated IC₅₀ values for each endometriosis organoid model following rimegepant treatment. Left to right: EO-30 (black), EO-47 (red), EO-32 (blue). **G-H)** Mean change in organoid area (%) from baseline (pre-treatment) in vehicle and ≈ IC₅₀ for each organoid model. Left to right: EO-30, EO-47, EO-32. Data represent the mean of replicate wells per timepoint and treatment, expressed relative to baseline (day 5, pre-treatment) ±SEM. Grey shading indicates treatment period. **F)**. Growth trajectories of three representative EO-30 organoids treated with 3.125 µM, imaged at 12-hour intervals from pre-treatment (–1 h) to 120 h post-treatment. Orange lines indicate treatment administration at 0 h and 72 h. Individual organoids displayed varied responses, including halted growth, gradual increase, and reduction in size over the treatment course (as indicated by arrows).

## Supplementary Methods

**1.1 Transcriptomic Analysis**

Processed RNA-sequencing data from triplicate vehicle-treated immortalised endometriotic epithelial 12Z cells were obtained from GSE148473 (Wilson et al., 2020). Normalised expression values for the target gene *ROR1* were extracted from the DESeq2-normalised counts file. Two reference genes, *TBP* and *HPRT1*, were selected as expression comparators based on their moderate expression and prior validation as suitable controls in endometrial and endometriosis transcriptomic studies (Vestergaard et al., 2011) .

**1.2 Western Blot**

Reference endometrial cancer cell lines RL95-2 and KLE were selected due to known expression of ROR1 at low to moderate and high levels, respectively (Liu et al., 2025a). RL95‐2 was a gift from Professor Deborah Marsh (UTS, Australia). 12Z, RL95-2 and KLE cell lines were maintained in medium containing 10% foetal bovine serum (Scientifix), 1% GlutaMAX (ThemoFisher) and 1% penicillin/streptomycin (ThemoFisher) and cultured in 5% CO_2_ at 37°. All cell lines were maintained in a base medium of DMEM/F12, and RL95‐2 was supplemented with 0.005 mg/mL insulin.

Total protein was extracted from the cells using cell lysis buffer (Cell Signalling Technology) with protease inhibitor (Sigma-Aldrich). 20 µg protein samples were separated on 8% polyacrylamide gels and transferred onto nitrocellulose membranes. 3% non-fat milk (Coles, Australia) in 0.1% Tween in Tris buffered saline (TBST) was used as blocking buffer and antibody diluent. The membranes were blocked for 1 h at room temperature before the overnight incubation with primary antibody at 4 °C. The primary antibodies used were monoclonal rabbit anti-ROR1 (#AF2000, R&D Systems, 1:500) and monoclonal mouse anti-GAPDH (ab8245, Abcam, 1:10,000). After washing with TBST, the membranes were incubated with either polyclonal rabbit anti-mouse immunoglobulins/HRP (#P0260, Dako, Denmark) or polyclonal rabbit anti-goat immunoglobulins/HRP (#P0449, Dako, Denmark) at 1:5,000 dilution for 1 h at room temperature. After another set of washes, the membranes were incubated with enhanced chemiluminescence reagents (ThermoFisher) and imaged using the Invitrogen iBright imaging system (ThermoFisher Scientific).
